# Species traits predict the aryl hydrocarbon receptor 1 (AHR1) subtypes responsible for dioxin sensitivity in birds

**DOI:** 10.1038/s41598-020-68497-y

**Published:** 2020-07-16

**Authors:** Kristin Bianchini, Christy A. Morrissey

**Affiliations:** 1 Long Point Waterfowl and Wetlands Research Program, Birds Canada, 115 Front Road, Port Rowan, ON N0E 1M0 Canada; 2 Biology Department, Acadia University, Wolfville, NS B4P 2R6 Canada; 3 Department of Biology, University of Saskatchewan, 112 Science Place, Saskatoon, SK S7N 5E2 Canada; 4 School of Environment and Sustainability, University of Saskatchewan, 117 Science Place, Saskatoon, SK S7N 5C8 Canada

**Keywords:** Conservation biology, Ecophysiology, Phylogenetics, Ecology

## Abstract

Differences in avian sensitivity to dioxin-like compounds (DLCs) are directly attributable to the identities of amino acids at two sites within the ligand binding domain (LBD) of the aryl hydrocarbon receptor 1 (AHR1). Recent work suggests that by influencing avian exposure to naturally occurring dioxins, differences in diet, habitat, and migration may have influenced the evolution of three AHR1 LBD genotypes in birds: type 1 (high sensitivity), type 2 (moderate sensitivity), and type 3 (low sensitivity). Using a boosted regression tree (BRT) analysis, we built on previous work by examining the relationship between a comprehensive set of 17 species traits, phylogeny, and the AHR1 LBD across 89 avian species. The 17 traits explained a combined 74% of the model deviance, while phylogenetic relatedness explained only 26%. The strongest predictors of AHR1 LBD were incubation period and habitat type. We found that type 3 birds tended to occupy aquatic habitats, and, uniquely, we also found that type 3 birds tended to have slower developmental rates. We speculate that this reflects higher evolutionary exposure to naturally occurring dioxins in waterbirds and species with K-selected life histories. This study highlights the value of trait-based approaches in helping to understand differing avian species sensitivities to environmental contaminants.

## Introduction

Dioxin-like compounds (DLCs) are environmental contaminants of great ecotoxicological concern. This family of pollutants includes the polychlorinated dibenzo-*p*-dioxins (PCDDs), polychlorinated dibenzofurans (PCDFs), and several polychlorinated biphenyls (PCBs)^1^. DLCs pose an important threat to avian wildlife due to their persistence and ubiquity in the environment and their known toxic effects in birds, which include developmental abnormalities, reproductive impairment, compromised immune function, and behavioural changes^1,2^. Indeed, DLC exposure has been associated with reproductive failures and population declines in many avian species, most notably in fish-eating birds in the North American Great Lakes^3^.

Most of the known toxic and biochemical effects of DLCs are mediated by their binding to the aryl hydrocarbon receptor (AHR)^4,5^. When DLCs or other ligands bind to the AHR, the AHR translocates to the nucleus, where it forms a heterodimer with the aryl hydrocarbon receptor translocator (ARNT). This interaction allows the AHR to bind the dioxin-responsive element, a promoter element of xenobiotic-metabolizing enzymes such as cytochrome P4501A (CYP1A). Ultimately, AHR activation mediates the toxic effects of DLC exposure^6,7^. In addition to its role in sensing environmental contaminants, recent evidence suggests that the AHR plays an integral role in cancer promotion, liver disease, and the function of the immune and nervous systems^6^. AHR-mediated signalling is present and highly evolutionarily conserved in a variety of organisms^8^, suggesting that the AHR has an important biological function^6,7^. However, the AHR is an orphan receptor, and its endogenous role is not well understood^7^. Although a variety of endogenous AHR ligands have been identified^6^, and there is evidence that exogenous factors may have influenced the evolution of the AHR^9^, the endogenous role of the AHR and the reason why it is so highly conserved are unknown^6,7^.

**Figure 1:**
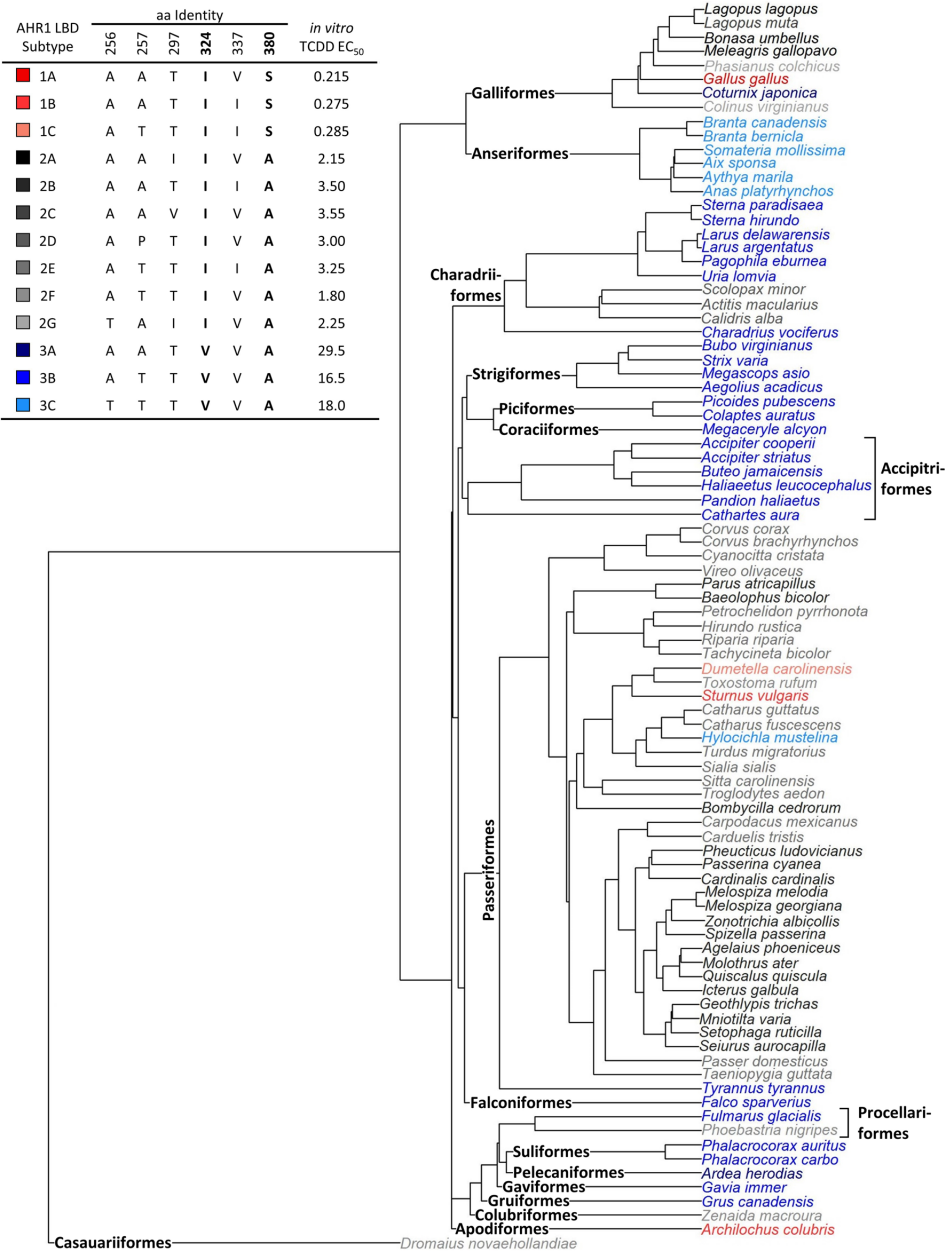
Phylogenetic distribution of the 13 known AHR1 LBD protein subtypes among 89 avian species. Font colors are indicative of the 13 AHR1 LBD protein subtypes, with red, grey, and blue representing the type 1 (high sensitivity, Ile324_Ser380), type 2 (moderate sensitivity, Ile324_Val380) and type 3 (low sensitivity, Val324_Ala380) protein subtypes, respectively. Avian orders are indicated on the phylogenetic tree in boldface type. Table inset shows the amino acid identities and in vitro TCDD EC_50_ values of each AHR1 LBD subtype identified by Farmahin et al.^11^.

Despite the evolutionary conservation of the AHR-mediated signalling pathway, avian species show high interspecies variation (up to 1,000-fold) in sensitivity to DLCs^10–12^. Previous work has established that differences in avian DLC sensitivity are directly related to the identity of amino acids at sites 324 and 380 within the ligand-binding domain (LBD) of the aryl hydrocarbon receptor 1 (AHR1)^10,11,13^, and differences in amino acid residues at other sites do not contribute to inter-species variations in DLC sensitivity^13^. It has been proposed that birds can be classified into three main groups based on the identities of amino acids at these two sites: type 1 (high sensitivity, Ile324_Ser380), type 2 (moderate sensitivity, Ile324_Val380), and type 3 (low sensitivity, Val324_Ala380)^10,13–15^. To date, sequence analyses have identified four other amino acid sites that are variable within the AHR1 LBD among avian species (sites 256, 257, 297, and 337), and the identities of amino acids at these sites have been used to further categorize the AHR1 into 13 LBD subtypes (see table inset in Fig. 1 for AHR1 LBD subtypes and their amino acid sequences)^11^.

Remarkably, there appears to be little correspondence between the three AHR1 LBD types and phylogeny, particularly for the high sensitivity type 1 group^9,16^. Hwang et al.^9^ showed that the transactivation potential of a naturally-occurring dioxin was in the order of type 1 > type 2 > type 3 and demonstrated that factors related to the exposure of birds to naturally occurring dioxins, including a species’ habitat and diet, may have contributed to the natural selection of the three AHR1 LBD types in avian species during their evolution. It is therefore possible that other biological and ecological factors could have influenced the evolution of the 13 AHR1 LBD subtypes in birds, but this has not been explored.

Here we present an exploratory analysis examining whether trait-based approaches, used more widely in ecology, can explain why certain birds have different AHR1 LBD subtypes than other phylogenetically related species. Species traits are quantifiable characteristics that reflect a species’ adaptation to its environment^17^, including physiological and ecological characteristics, like a species’ body size, longevity, reproductive output, and habitat^18^. Trait-based approaches have shown tremendous potential to explain functional patterns in ecological communities and to predict physiological responses to environmental stressors^19^. However, the use of traits in ecotoxicological research is relatively limited to invertebrate species^20^.

Our objective was to evaluate support for a broad suite of species traits that might explain variations in the 13 AHR1 LBD subtypes in birds. We collected data for the three traits examined by Hwang et al.^9^ (diet, habitat, and migration), and we built on their work by also collecting information for the maximum number of physiological and ecological traits for which there were sufficient data for a large number of avian species. Ultimately, we examined traits indicative of each species’ developmental rate, fecundity and reproduction, levels of contaminant depuration into the egg, body size, and longevity (physiological traits), and each species’ migration strategy, range, habitat type, tropic level, and degree of sociality and sexual competition (ecological traits). Although we did not form a priori assumptions as to how these traits might affect species sensitivity to DLCs, migration strategy, range, habitat, foraging guild, and the level of depuration into the egg can affect a species’ level of toxicant exposure^21,22^, and fecundity, the degree of sociality and sexual competition, body size, lifespan, developmental rate, and migration strategy can affect a species’ susceptibility to population decline and a population’s ability to recovery following environmental disturbances, like a contamination event^21,23–26^. Our exploratory analysis used a novel analytical method to examine the association between these 17 species traits, phylogenetic relatedness, and the 13 avian AHR1 LBD subtypes across 89 avian species. Given recent evidence that some species traits were predictive of the three AHR1 types in birds^9^, we hypothesized that avian species traits are stronger correlates of the 13 AHR1 LBD subtypes than phylogeny.

Our analysis used in vitro TCDD EC_50_ values as a continuous proxy for the 13 known AHR1 LBD subtypes in birds, as using a continuous response allowed for more flexible statistical analyses that could more thoroughly explore species traits as sources of variation in the 13 AHR1 LBD subtypes in birds. The in vitro TCDD EC_50_ values used here represent the level of AHR1-mediated CYP1A induction in a cell line transfected with the avian AHR1 constructs following TCDD exposure^11^, and these values are strongly correlated with *in ovo* LD_50_ values^11^. However, it should be noted that although TCDD EC_50_ is a strong predictor of avian in vivo sensitivity^12^, in vitro TCDD EC_50_ values only account for the portion of in vivo sensitivity than can be explained by the sequence of the AHR1 LBD, and other molecular mechanisms (e.g., other domains of the AHR, cofactors, downstream responses^13^), and individual factors (e.g., age and sex^27^) can also contribute to inter-species differences in in vivo sensitivity.

## Results

### Phylogenetic distribution of the AHR1 LBD

To understand the phylogenetic distribution of the AHR1 LBD amino acid subtypes, we visualized the AHR1 LBD subtype of the 89 avian species within a phylogenetic tree (Fig. 1). The high sensitivity type 1 AHR1 LBD (Ile324_Ser380) showed strong phylogenetic divergence, and was expressed in < 5% (4/89) of the available study species, including one Galliformes species (Red Jungle Fowl, *Gallus gallus*; subtype 1A), two Passeriformes species (European Starling, *Sturnus vulgaris*, subtype 1B; and Gray Catbird, *Dumetella carolinensis*, subtype 1C), and one Apodiformes species (Ruby-throated Hummingbird, *Archilochus colubris*; subtype 1B). In contrast, the type 2 and 3 AHR1 LBDs dominated in certain avian orders. Most species in our dataset (55%) expressed the moderate sensitivity type 2 AHR1 LBD (Ile324_Val380). The type 2 AHR1 LBD was dominant among the Galliformes (here, predominantly the Phasianidae family, which expressed subtypes 2A, 2C, and 2G), the Scolopacidae of the Charadriiformes shorebirds (all subtype 2D), and the Passeriformes (subtypes 2B and 2E). Thirty-six of the 89 avian species (40%) expressed the low sensitivity type 3 AHR1 LBD (Val324_Ala380), and the majority of these low sensitivity species (75%) expressed the 3B subtype. The type 3 AHR1 LBD was dominant in the remaining Chardriiformes species (subtype 3B), the Strigiformes (subtype 3B), the Accipitriformes (subtype 3B), and the Anseriformes (subtype 3C), which in this dataset were predominantly represented by the Laridae, Strigidae, Accipitridae, and Anatidae families, respectively.

### Relationship between species traits, phylogeny, and AHR1 LBD protein subtypes

**Figure 2:**
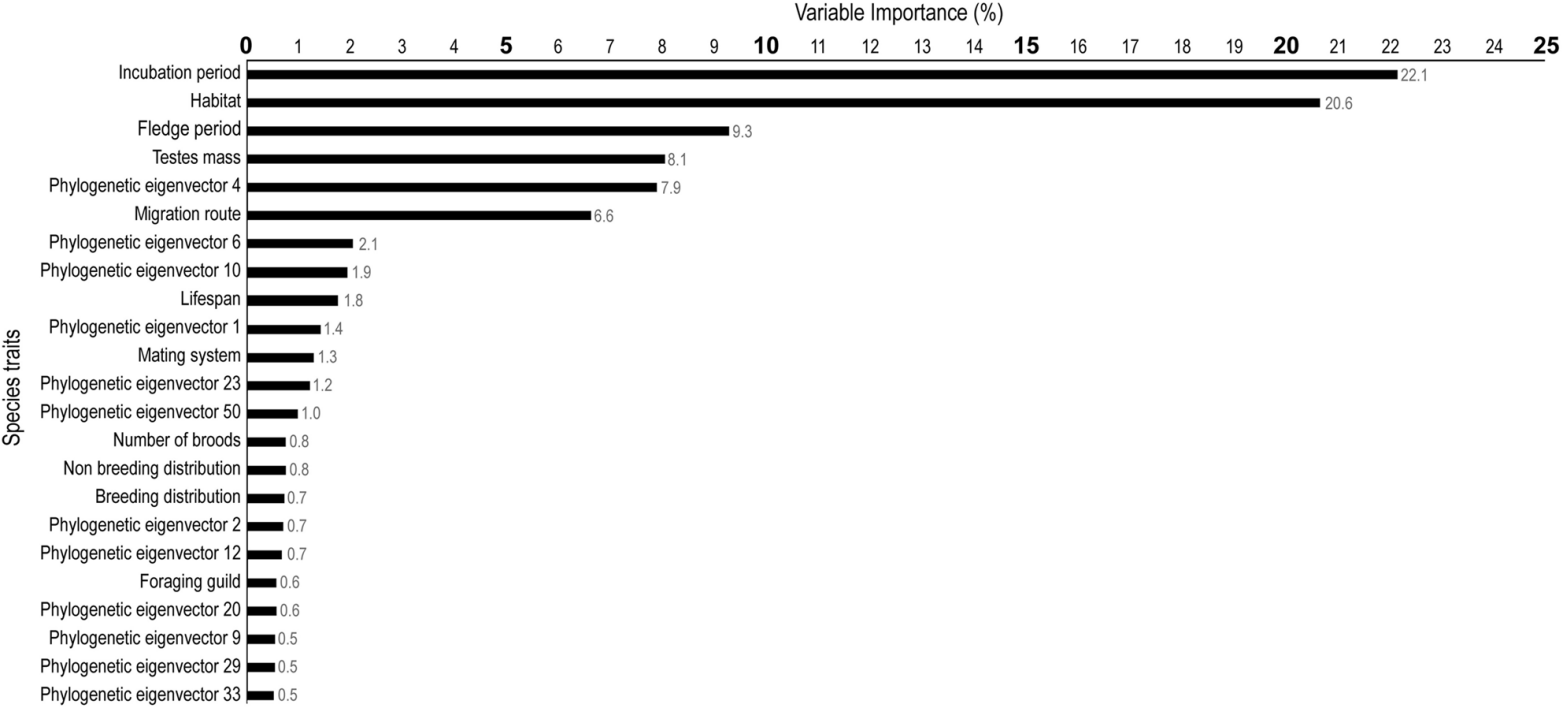
Species traits and measures of phylogenetic relatedness identified as the most important for predicting TCDD EC_50_. Variable importance (VI) scores indicate the proportion of the model deviance explained by that variable. The sum of VI scores is equal to 100. Only variables with a VI score ≥ 0.5% are depicted.

We used boosted regression trees (BRTs) to simultaneously rank measures of phylogenetic relatedness (i.e., phylogenetic eigenvectors) and species traits as correlates of the AHR1 LBD. In our analysis, in vitro TCDD EC_50_ values were used as a continuous proxy for the 13 known AHR1 LBD protein subtypes. The BRT explained 73.8% of the deviance in TCDD EC_50_. The 17 species traits explained a combined 74.0% of the model deviance, while all phylogenetic eigenvectors explained a combined 26.0% of the model deviance (i.e., species traits and phylogeny explained 54.6% and 19.2% of the deviance in TCDD EC_50_, respectively; their sum = 73.8%). The strongest predictors of TCDD EC_50_ were incubation period (variable importance (VI) score = 22.1%, indicating that incubation period explained 22.1% of the model deviance) and habitat type (explained 20.6% of the model deviance). Other variables of lesser importance were fledge period (9.3%), testes mass (8.1%), phylogenetic eigenvector 4 (7.9%), and migration route (6.6%; Fig. 2; the VI scores of all variables included in the BRT are shown in Supplementary Table S1).

**Figure 3:**
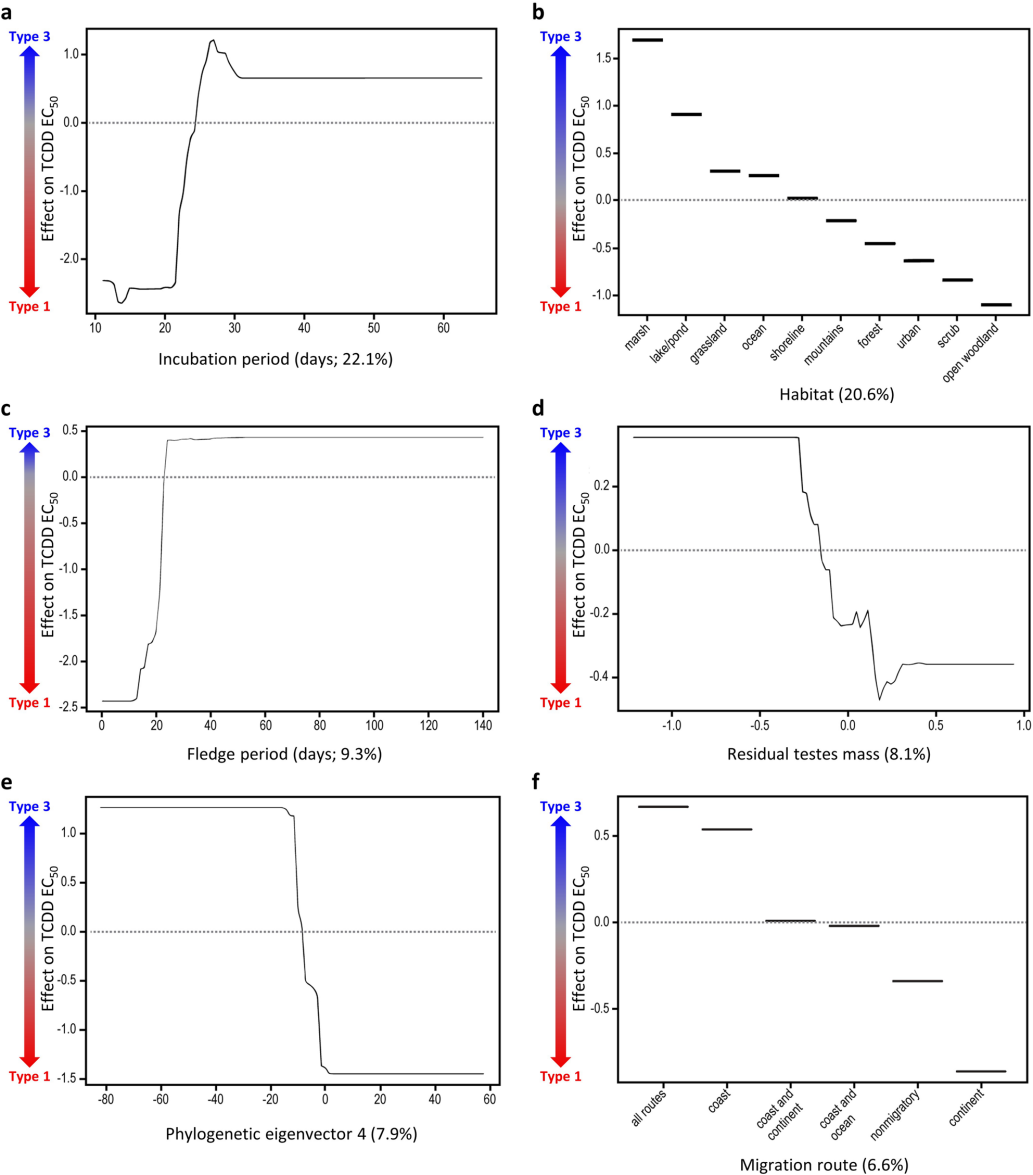
The relationship between incubation period (**a**), habitat (**b**), fledge period (**c**), residual testes mass (**d**), phylogenetic eigenvector 4 (**e**), migration route (**f**), and in vitro TCDD EC_50_ (shown here as a dimensionless transformation), where more negative TCDD EC_50_ values indicate that a species is more likely to express the type 1 AHR1 LBD (high sensitivity, Ile324_Ser380; indicated by the red arrow), and more positive TCDD EC_50_ values indicate that a species is more likely to express the type 3 AHR1 LBD (low sensitivity, Val324_Ala380; indicated by the blue arrow). Variable importance (VI) scores (i.e., the proportion of the model deviance explained by each predictor) are indicated in parentheses in the x-axis labels.

We further explored the relationship between the top six predictor traits and in vitro TCDD EC_50_, where a negative effect on TCDD EC_50_ indicates a trend towards the high DLC sensitivity type 1 AHR1 LBD, and a positive effect on TCDD EC_50_ indicates a trend towards the low DLC sensitivity type 3 AHR1 LBD. TCDD EC_50_ increased with incubation period, indicating that type 3 (low sensitivity) birds tended to have protracted incubation periods, reflecting a longer developmental time in the egg (Fig. 3a). We found that type 1 (high sensitivity) birds were more likely to use open woodland and scrub habitats. Conversely, type 3 birds were more likely to inhabit lake/pond and marsh habitats (Fig. 3b). We also found that type 1 birds were more likely to have shorter fledge periods (Fig. 3c), lower testes masses (indicating lower sexual competition; Fig. 3d), more negative values along phylogenetic eigenvector 4 (Fig. 3e), and to use continental (inland) migration routes or to be non-migratory (Fig. 3f). There were no significant interactions among the predictor variables examined.

## Discussion

This study presents an exploratory trait-based analysis examining the relationship between 17 biological and ecological traits, phylogeny, and the 13 avian AHR1 LBD subtypes across a large sample of 89 bird species. We used novel analytical methods that included 3 previously identified traits of importance with respect to the evolution of the avian AHR, as reported by Hwang et al.^9^, and 14 additional species traits. Our results support that previous work, showing the importance of habitat as a predictor of the AHR1 LBD subtype in avian species, and to a lesser extent migration distance. We also identified incubation period as a strong correlate of avian AHR1 LBD subtypes. Our study therefore builds on the findings of previous research and generates new hypotheses as to the functional roles of species traits in the evolution of the AHR in birds.

The distribution of the 13 AHR1 LBD subtypes on an avian phylogenetic tree revealed that the type 2 (moderate sensitivity) and type 3 (low sensitivity) AHR1 LBD subtypes dominated in certain avian orders, but that type 1 (high sensitivity) was poorly predicted by relatedness. The distribution of type 2 and 3 AHR1 LBD types corresponded with our finding that habitat was a stronger predictor of TCDD EC_50_. The moderate sensitivity type 2 AHR1 LBD was dominant among Passeriformes (subtypes 2B and 2E) and Galliformes (subtypes 2A, 2C, and 2G), which tend to occupy terrestrial habitats, whereas the low sensitivity type 3 AHR1 LBD was dominant among birds in aquatic habitats, such as the Anseriformes (subtype 3B). Hwang et al.^9^ also found that a species’ diet was an important predictor of its AHR1 genotype. Indeed, we saw a predominance of the type 3 AHR1 LBD among carnivorous and piscivorous avian orders (e.g., Accipitriformes, Falconiformes, Stirgiformes), but in our analysis, diet did not receive significant statistical support (foraging guild only explained 0.6% of the model variance). This result is unexpected, as one would predict that higher levels of dioxin exposure in higher trophic level species would have favoured the evolution of the low sensitivity, type 3 AHR LBD^9^. Instead, we saw a broad distribution of the type 3 AHR1 LBD among birds from diverse foraging guilds. Indeed, of the 36 birds with the type 3 AHR1 LBD, nearly half (42%) were omnivorous, herbivorous, or invertivorous (e.g., birds in the Piciformes, Anseriformes, Passeriformes orders). However, many factors, in addition to a species’ foraging guild, can influence its exposure to and bioaccumulation of contaminants (e.g., body size, sex, habitat conditions, migration, lipid content)^28^. Likewise, an individual’s trophic position is influenced by complex ecological processes, such as a community’s food chain length^29^. Therefore, further research will be needed to explain these conflicting results and to clarify how diet and/or trophic position may be associated with the AHR1 LBD.

Nevertheless, our study provides support for the finding that different histories of contamination and exposure to DLCs across habitats may have shaped the evolution of the AHR1 LBD. We found that birds in aquatic habitats tended to have the least sensitive, type 3 AHR1 LBD and that birds in terrestrial environments tended to have the more sensitive type 1 AHR1 LBD. Exposure to naturally occurring dioxins may have exerted differential selective pressure on birds, which generated the three AHR1 genotypes in avian species^9^. Naturally occurring dioxins have been identified in aquatic environments (e.g., polybrominated dibenzo-*p*-dioxins (PBDDs), which are synthesized in red algae and have been found in high concentrations in aquatic biota)^30,31^ and terrestrial environments (e.g., 1,3,6,8- and 1,3,7,9-tetrachlorodibenzo-*p*-dioxins and 2,4,6,8-tetracholorodibenzofuran, which are produced in slime molds and lichens)^32–34^. Moreover, naturally occurring polycyclic aromatic hydrocarbons (PAHs), which are present in crude oil^35^ and produced by the incomplete combustion of organic matter (e.g., wood burning^36,37^), can also bind the AHR and elicit AHR-mediated toxicities^38^. However, PAHs also have other, non-AHR-mediated toxic mechanisms of action (e.g., biotransformation to reactive metabolites^39^). Aquatic environments are often acknowledged to have higher contaminant levels than terrestrial environments. This is because terrestrial contaminants are washed into freshwater environments and are ultimately transported to the marine environment, which is a sink for contaminants globally^40,41^. Indeed, concentrations of naturally occurring PBDDs in aquatic biota exceeded the European Commission’s maximum residue limits of 4 pg TCDD equivalents (TEQ)/g fish muscle^30,31^, suggesting that concentrations of naturally occurring dioxins may have been high enough to cause toxicity and selective pressure on avian species. Our study suggests that birds evolving in aquatic environments may have been exposed to higher levels of naturally occurring dioxins and possibly anthropogenic sources. Higher exposure could act as selection pressure for a lower dioxin binding affinity to help mitigate dioxin toxicity. It is unknown what AHR1 LBD variants were present in avian evolutionary history, and additional research will be needed to determine how habitat and higher historical DLC exposures could influence the evolution of the 13 AHR1 LBD subtypes in birds. As well, if high DLC exposure affected the evolutionary history of the AHR, it is possible that more recent avian population bottlenecks (e.g., dramatic declines in piscivorous bird populations in the 1960s and 1970s due to high DLC concentrations in the Great Lakes^3^) may have similarly affected dioxin binding affinity and the AHR1 LBD in certain avian populations, and this could be an interesting avenue for future ecotoxicological research.

Our visualization of the phylogenetic distribution of the 13 AHR1 LBD subtypes also revealed that the high sensitivity type 1 AHR1 LBD showed the strongest phylogenetic divergence. However, the type 1 genotype was found in only four of our 89 species, suggesting that the type 1 AHR1 LBD is relatively rare in birds^9^. Although more research is needed to identify whether the type 1 AHR1 LBD is associated with phenotypes other than DLC sensitivity, the low number of highly sensitive birds suggests that possession of the type 1 AHR1 LBD may be disadvantageous or it may confer a selective advantage to a small number of species^9^.

A novel finding of our study was that incubation period was the strongest correlate of TCDD EC_50_. Incubation period represents the growth rate of avian species before hatch, and our results suggest that species with slower developmental times tended to have the least sensitive type 3 AHR1 LBD. The in vitro TCDD EC_50_ values that we used for analysis are highly correlated (*r*^2^ = 0.95) with *in ovo* LD_50_ values^11^, suggesting that our response variable is strongly predictive of the lethality of TCDD to birds in early development. This may explain why a species trait related to avian embryonic development explained the majority of the variation in TCDD EC_50_. Although, we acknowledge that the in vivo sensitivity of most adult birds is unknown.

It is difficult, however, to draw a direct link between variations in incubation period and the evolution of different AHR1 LBD sequences. It is possible that longer developmental times reflect a tendency towards a K-selected life history. Combined, the nine species traits indicative of r/K trade-off strategies (i.e., broods per year, clutch size, fledge period, incubation period, stage at hatch, lifespan, body mass, percent of female body mass represented by the clutch, and testes mass) explained 43% of the variance in TCDD EC_50_, and type 3 birds tended to exhibit more of the characteristics of K-selected species. On average, birds with the type 3 AHR1 LBD had longer lifespans, longer developmental times, larger body sizes, and lower reproductive investment than the type 1 and 2 birds in this dataset (see Supplementary Table S2). We propose it is possible that the traits associated with a K-selected life history may increase a bird’s exposure to naturally occurring dioxins^42^. Longer lifespans, larger body sizes, and slower metabolic rates are associated with higher contaminant accumulation and slower clearance. Likewise, large per capita investment in fewer offspring is associated with high maternal depuration into the egg, and long embryonic (i.e., incubation) periods, during which offspring are sustained by the yolk, prolong the exposure of offspring to maternally derived contaminants during development. It is therefore possible that our results reflect the higher exposure of K-selected species to naturally occurring dioxins during avian evolution. Higher dioxin exposure could have exerted additional selective pressure on these K-selected birds and favoured selection of the less sensitive type 3 AHR1 LBD as they evolved. Further work will be needed to fully confirm this hypothesis; however, it provides an insight for ecotoxicologists that are interested in predicting species specific risk.

Our study provides evidence supporting the use of trait-based approaches in avian ecotoxicology. Traditional toxicological testing and risk assessment methods assume that related species share similar xenobiotic sensitivities and extrapolate toxicity data from one species to another based on phylogenetic relatedness^43^. Our results lend support to a growing body of evidence that this is likely an imprecise practice for DLCs, given that AHR1 LBD subtypes, which are strong predictors of avian DLC sensitivity^10,11,13^, do not correspond well with avian phylogeny^9,16^. Our study illustrates the value of trait-based approaches to better understand the evolution of factors that affect species sensitivity with application for conservation. We also show how species traits can increase our understanding of toxicant exposure outcomes in species that share similar life histories. Ultimately, trait-based approaches provide a complementary method to help conservation practitioners prioritize groups of birds that are more vulnerable to current and future threats from environmental contaminants.

## Methods

### Data collection

Our analysis used in vitro TCDD EC_50_ values as a continuous proxy for the 13 known AHR1 LBD subtypes in birds. The AHR1 LBD amino acid sequences of 89 avian species and their associated in vitro TCDD EC_50_ values were obtained from Farmahin et al.^11^, who transfected each of the 13 AHR1 LBD subtypes into a COS-7 cell line, treated cells with TCDD, and measured AHR1 activation levels using a Luciferase Reporter Gene assay^14^. Their resulting EC_50_ values were strongly positively correlated (*r*^2^ = 0.93) with egg injection (*in ovo*) median lethal dose (LD_50_) values, suggesting the in vitro assays are predictive of the avian embryonic response to DLCs.

We also collected data for 17 species traits. Traits were broadly selected to explore the physiological and ecological life histories of different species, including developmental rate, fecundity, level of contaminant depuration into the egg, body size, longevity, migration strategy, range, habitat type, trophic level, and degree of sociality and sexual competition (Table 1). Unless otherwise indicated, trait data were obtained from The Birds of North America^44^.

**Table 1:** Description of the species traits and representative species included in the analysis.

Trait category	Species trait	Trait levels/range	Representative species
Developmental rate	Incubation period	Min: 11 days Max: 65.6 days	Chipping sparrow Black-footed albatross
	Fledge period	Min: 0.08 days Max: 140 days	Spotted sandpiper Black-footed albatross
	Stage at hatch	Precocial Altricial	Sanderling Tree swallow
Fecundity	Clutch size	Min: 1 egg Max: 14 eggs	Thick-billed murre Bobwhite quail
	Broods per year	Min: 1 brood Max: 3.5 broods	Black-capped chickadee Mourning dove
Body size	Body mass	Min: 3.4 g Max: 34,200 g	Ruby-throated hummingbird Emu
Level of depuration into egg & relative female investment	% of female body mass represented by clutch	Min: 3.1% Max: 95%	Red-winged blackbird Spotted sandpiper
Migration strategy	Migration route(s)	Non-migratory Continental Coastal Continental and coastal Coastal and oceanic All routes	Ring-necked pheasant European starling Belted kingfisher Song sparrow Herring gull Wood thrush
	Migration distance	Min: 0 km Max: 113.5 km	Downy woodpecker Arctic tern
Species range	Breeding range	Very widespread Widespread Intermediate Local Highly restricted	Common flicker Common loon Common tern Sanderling Black-footed albatross
	Wintering range	Very widespread Widespread Intermediate Local Highly restricted	House finch Hermit thrush Tufted titmouse Common eider No examples in this dataset
Degree of sociality and sexual competition	Testes mass*	Min: − 1.22 Max: 0.95	Greater scaup Red jungle fowl
	Social mating system	Polyandrous Monogamous Mostly monogamous Polygynous Lekking/promiscuous Cooperative breeders	Spotted sandpiper Ovenbird Barn swallow Red-winged blackbird Ruffed grouse No examples in this dataset
	Breeding coloniality	Solitary Semi-colonial Colonial	Northern cardinal Common grackle Ring-billed gull
Habitat type	Habitat	Urban Forest Grassland Lake/pond Marsh Mountain Ocean Open woodland Scrub Shoreline	House sparrow American redstart Eastern bluebird Osprey Great blue heron Northern raven Ivory gull Turkey vulture Brown thrasher Common tern
Trophic level	Foraging guild	Carnivorous Herbivorous Insectivorous Invertivorous Omnivorous Piscivorous	Barred owl American goldfinch Bank swallow Killdeer Blue jay Great cormorant
Longevity	Lifespan	Min: 2.25 years Max: 40 years	Japanese quail Black-footed albatross

*Calculated as residual testes mass to correct for body size. Higher testes mass = higher sexual competition.

We collected data for three species traits indicative of developmental rate: incubation period, fledge period (number of days between hatching and fledging), and stage at hatch (altricial or precocial, entered as unordered categories). Average clutch size (from Pitcher et al.^45^) and number of broods per year were used as proxies of annual fecundity. The potential level of depuration into the egg was estimated by calculating the percentage of the female body mass represented by the clutch ((clutch size × egg mass)/adult mass, excluding renesting; from Robinson et al.^46^). We used average body mass (from Dunning^47^) as a measure of a species’ body size, and longevity was estimated from the average lifespan of each species in the wild.

Migration strategy variables included migration routes and migration distance. Migration routes were divided into six unordered categories (non-migratory, continental, coastal, continental and coastal, coastal and oceanic, all routes). Migration distance was calculated as the difference in degrees latitude between the midpoint of a species’ breeding range and the midpoint of a species’ wintering range^26^. Range midpoints were approximated as the median latitude between the most northerly and southerly range extents of each species’ breeding and wintering occurrences (median latitudes were estimated using Google Maps^48^). Range was determined by each species’ breeding and wintering range. Breeding and wintering ranges were scored as ordered categories according to the system used in Thomas et al.^26^, which approximates the geographical area occupied by a species in a particular season: 1 = very widespread, 2 = widespread, 3 = intermediate, 4 = local, and 5 = highly restricted.

Species habitats were categorized as urban, forest, grassland, lake/pond, marsh, mountain, ocean, open woodland, scrub, and shoreline (unordered categories). Trophic levels were inferred from each species foraging guild, which were classified as carnivorous, herbivorous, insectivorous, invertivorous, omnivorous, and piscivorous (unordered categories). Data on the degree of social interactions and type of sexual selection were represented by testes mass, social mating system, and breeding coloniality (obtained from Pitcher et al.^45^). Testes mass was corrected for body size using the residuals from a regression of testes mass on body mass. Species were separated into six unordered mating system categories (originally determined from behavioral analyses): 1 = polyandrous; 2 = monogamous (< 5% polygyny); 3 = mostly monogamous, occasionally polygynous (5–15% polygyny); 4 = polygynous (> 15% polygyny); 5 = lekking or promiscuous; 6 = cooperative breeder. Breeding coloniality was divided into three ordered categories: 0 = solitary, 1 = semi-colonial, 2 = colonial.

### Phylogenetic reconstruction

Phylogenetic relatedness among the 89 avian species was determined using the methods outlined in Rubolini et al.^49^. Briefly, 1,000 randomly sampled post burn-in phylogenetic trees were downloaded from “BirdTree” (www.birdtree.org)^50,51^ using the Hackett et al.^52^ backbone phylogeny. This tree set was summarized into a 50% majority-rule consensus tree (described in Holder et al.^53^; Rubolini et al.^49^) using the “SumTrees” program^54^ (version 4.0.0), which is part of the “DendroPy” phylogenetic computing library^55^ (version 4.0.3), and was run using Python 3.5.1^56^. The resulting phylogenetic tree was plotted using the ape^57^ and picante^58^ packages in R version 3.2.2^59^.

### Statistical analysis

Statistical analysis was performed using boosted regression trees (BRTs). The assumptions of other widely used models (e.g., phylogenetic generalized least squares (PGLS) models^60^) require the exclusion or conversion of variables with a high degree of collinearity, as found among many of our predictor variables. BRTs are a machine-learning method that circumvents this issue by combining the strengths of two algorithms, regression trees and boosting, to fit a single parsimonious model. Regression trees are advantageous for analyzing datasets with numerous predictors because they can model linear and nonlinear relationships between categorical and numeric predictors, they automatically handle any complex interactions and missing values, and they are relatively insensitive to collinearity^61,62^. Boosting lends predictive power to this method by building and combining a large number of these individual regression trees in a forward, stepwise fashion^61,62^. BRTs thus have superior predictive performance for datasets with a large number of predictors, because these algorithms develop a model from the data by “learning” the relationship between a response and predictors and thus avoid using a predetermined model^62^. BRTs are also advantageous in that they summarize complex relationships and interactions using simple graphical and numerical approaches^61,62^.

Avian species are not statistically independent because species with common ancestors are more likely to exhibit similar traits. This is known as ‘phylogenetic non-independence’ and must be controlled for in comparative studies^63–65^. There is as of yet no automated program for directly including measures of phylogenetic relatedness into BRTs. We therefore used a phylogenetic eigenvector regression (PVR) approach to account for phylogenetic non-independence^66^. To do this, we computed a phylogenetic distance matrix from our above 50% majority-rule consensus tree (see Supplementary Data S1) and extracted eigenvectors from this distance matrix using a principal coordinate analysis (see Supplementary Data S2; performed using ape package^57^). These eigenvectors represent the phylogenetic relationship among species in a vector form (eigenvectors and their usage are described in Diniz-Filho et al.^67^).

The PVR approach is a reliable statistical method for phylogenetic inclusion in the BRT, and this method has been used successfully to address numerous questions in a diversity of taxa (e.g.,^68–72^). Studies estimating correlations among traits using various phylogenetic comparative methods show that PVR has good (i.e. low) type I and II error rates and provides comparable results to other methods^69,73,74^. By not assuming the evolutionary model *a priori*^75^, the PVR provides a more robust and flexible method in instances where the true evolutionary model is complex or unknown, and the PVR has comparable statistical performance even under evolutionary processes that diverge from Brownian motion^69,71^. Furthermore, Diniz-Filho et al.^74^ demonstrated that phylogenetic eigenvectors accurately represent the phylogenetic relationships among species and control for phylogenetic autocorrelation when a sufficiently high number of phylogenetic eigenvectors (explaining at least 95% of the variation in the phylogenetic distances) are included in the analysis. For this reason, the first 53 eigenvectors, which explained 99% of the phylogenetic structure in the distance matrix (see Supplementary Table S3), were included as covariates in our BRT.

The relative importance or weighting of the predictors as correlates of TCDD EC_50_ are shown as variable importance (VI) scores. VI scores are calculated based on how often the variable is present in the regression tree set, weighted by the extent by which the variable improves the model fit, and averaged across the full model. The relative importance of each covariate is scaled so that all VI scores sum to 100, wherein higher VI scores indicate a greater correlation with TCDD EC_50_^62^. BRT analyses were conducted in package dismo^76^ in R version 3.2.2^59^.

## Supplementary information

Supplementary Information 1.

Supplementary Information 2.

Supplementary Information 3.

## Data Availability

Complete dataset available at the Federated Research Data Repository (FRDR): 10.20383/101.0257
