# Supplementary material for: Species traits predict the aryl hydrocarbon receptor 1 (AHR1) subtypes responsible for dioxin sensitivity in birds
Source: Sci Rep. 2020 Jul 16;10:11706. doi: 10.1038/s41598-020-68497-y (PMC7367299; doi:10.1038/s41598-020-68497-y)
Supplement: Supplementary file 3 — Supplementary Information 3. [file 41598_2020_68497_MOESM3_ESM.docx]

**Supplementary materials**

**Species traits** **predict the aryl hydrocarbon receptor 1 (AHR1) subtypes responsible for dioxin sensitivity in birds**

Kristin Bianchini^1^ & Christy A. Morrissey^2,3,*^

^1^Toxicology Centre, University of Saskatchewan, Saskatoon SK, S7N 5B3 Canada

^2^ Department of Biology, University of Saskatchewan, Saskatoon, SK, S7N 5E2 Canada

^3^ School of Environment and Sustainability, University of Saskatchewan, Saskatoon, SK, S7N 5C8 Canada

*christy.morrissey@usask.ca

**Table S1.** Variable importance (VI) scores (i.e., proportion of the model deviance explained by that variable) of all species traits and phylogenetic eigenvectors included in the BRT. The sum of VI scores is equal to 100.

| Variable | VI score (%) |
| --- | --- |
| Incubation period | 22.1 |
| Habitat | 20.6 |
| Fledge period | 9.28 |
| Residual testes mass | 8.06 |
| Phylogenetic eigenvector 4 | 7.88 |
| Migration route | 6.63 |
| Phylogenetic eigenvector 6 | 2.06 |
| Phylogenetic eigenvector 10 | 1.94 |
| Lifespan | 1.76 |
| Phylogenetic eigenvector 1 | 1.43 |
| Social mating system | 1.30 |
| Phylogenetic eigenvector 23 | 1.22 |
| Phylogenetic eigenvector 50 | 0.99 |
| Broods per year | 0.76 |
| Wintering range | 0.76 |
| Breeding range | 0.73 |
| Phylogenetic eigenvector 2 | 0.71 |
| Phylogenetic eigenvector 12 | 0.67 |
| Foraging guild | 0.58 |
| Phylogenetic eigenvector 20 | 0.57 |
| Phylogenetic eigenvector 9 | 0.54 |
| Phylogenetic eigenvector 29 | 0.54 |
| Phylogenetic eigenvector 33 | 0.53 |
| Mass | 0.47 |
| Phylogenetic eigenvector 43 | 0.46 |
| Breeding coloniality | 0.38 |
| Phylogenetic eigenvector 46 | 0.37 |
| Phylogenetic eigenvector 39 | 0.36 |
| Phylogenetic eigenvector 15 | 0.35 |
| Phylogenetic eigenvector 26 | 0.35 |
| Phylogenetic eigenvector 18 | 0.35 |
| Phylogenetic eigenvector 13 | 0.33 |
| Phylogenetic eigenvector 7 | 0.32 |
| Phylogenetic eigenvector 17 | 0.32 |
| Phylogenetic eigenvector 16 | 0.31 |
| Phylogenetic eigenvector 5 | 0.28 |
| Phylogenetic eigenvector 8 | 0.27 |
| Phylogenetic eigenvector 37 | 0.27 |
| Phylogenetic eigenvector 44 | 0.22 |
| Phylogenetic eigenvector 14 | 0.21 |
| Clutch size | 0.20 |
| Phylogenetic eigenvector 11 | 0.19 |
| Phylogenetic eigenvector 19 | 0.19 |
| Phylogenetic eigenvector 53 | 0.17 |
| Phylogenetic eigenvector 51 | 0.17 |
| Migration distance | 0.16 |
| Phylogenetic eigenvector 22 | 0.14 |
| Phylogenetic eigenvector 45 | 0.14 |
| Phylogenetic eigenvector 21 | 0.11 |
| Phylogenetic eigenvector 38 | 0.10 |
| % of female body mass represented by clutch | 0.10 |
| Phylogenetic eigenvector 31 | 0.10 |
| Phylogenetic eigenvector 41 | 0.10 |
| Phylogenetic eigenvector 3 | 0.09 |
| Phylogenetic eigenvector 28 | 0.09 |
| Phylogenetic eigenvector 24 | 0.09 |
| Phylogenetic eigenvector 42 | 0.08 |
| Phylogenetic eigenvector 27 | 0.08 |
| Phylogenetic eigenvector 40 | 0.05 |
| Phylogenetic eigenvector 49 | 0.05 |
| Stage at hatch | 0.05 |
| Phylogenetic eigenvector 34 | 0.05 |
| Phylogenetic eigenvector 32 | 0.05 |
| Phylogenetic eigenvector 30 | 0.04 |
| Phylogenetic eigenvector 47 | 0.04 |
| Phylogenetic eigenvector 36 | 0.04 |
| Phylogenetic eigenvector 52 | 0.01 |

**Table S2.** Summary statistics (mean ± s.d.) of species traits for birds with the type 1 (high sensitivity, Ile324_Ser380), type 2 (moderate sensitivity, Ile324_Val380), and type 3 (low sensitivity, Val324_Ala380) AHR1 LBD.

| Species trait | AHR1 LBD genotype | | |
| --- | --- | --- | --- |
|  | Type 1  (n = 4) | Type 2  (n = 49) | Type 3  (n = 36) |
| Incubation period | 12.7 ± 0.5 | 16.9 ± 10.3 | 27.3 ± 7.2 |
| Fledge period | 17.3 ± 6.0 | 16.0 ± 20.7 | 38.6 ± 18.7 |
| Clutch size | 4.3 ± 1.8 | 5.3 ± 2.6 | 4.1 ± 2.5 |
| Broods per year | 2.0 ± 0.0 | 1.6 ± 0.7 | 1.1 ± 0.3 |
| Body mass* | 257 ± 433 | 318 ± 992 | 1159 ± 1381 |
| % female body mass represented by clutch | 34.3 ± 1.9 | 39.9 ± 19.4 | 28.6 ± 19.5 |
| Migration distance | 1728 ± 1270 | 2022 ± 2326 | 1963 ± 2438 |
| Testes mass | 0.19 ± 0.58 | 0.17 ± 0.27 | -0.06 ± 0.53 |
| Lifespan | 14.1 ± 5.0 | 11.6 ± 6.1 | 20.2 ± 9.8 |

*Emu removed from mean body mass calculation for Type 2 birds (Emu body mass = 34,200 g).

**Table S3.** Total and cumulative proportion of the variance in the phylogenetic distance matrix explained by the phylogenetic eigenvectors.

| Phylogenetic eigenvector | Total variance explained | Cumulative proportion of variance explained |
| --- | --- | --- |
| 1 | 0.27 | 0.27 |
| 2 | 0.14 | 0.41 |
| 3 | 0.078 | 0.49 |
| 4 | 0.066 | 0.56 |
| 5 | 0.065 | 0.62 |
| 6 | 0.042 | 0.66 |
| 7 | 0.038 | 0.70 |
| 8 | 0.031 | 0.73 |
| 9 | 0.024 | 0.76 |
| 10 | 0.018 | 0.78 |
| 11 | 0.018 | 0.79 |
| 12 | 0.014 | 0.81 |
| 13 | 0.014 | 0.82 |
| 14 | 0.012 | 0.83 |
| 15 | 0.011 | 0.84 |
| 16 | 0.011 | 0.86 |
| 17 | 0.011 | 0.87 |
| 18 | 0.0095 | 0.88 |
| 19 | 0.0094 | 0.88 |
| 20 | 0.0092 | 0.89 |
| 21 | 0.0087 | 0.90 |
| 22 | 0.0083 | 0.91 |
| 23 | 0.0077 | 0.92 |
| 24 | 0.0075 | 0.93 |
| 25 | 0.0066 | 0.93 |
| 26 | 0.0050 | 0.94 |
| 27 | 0.0048 | 0.94 |
| 28 | 0.0037 | 0.95 |
| 29 | 0.0036 | 0.95 |
| 30 | 0.0036 | 0.95 |
| 31 | 0.0031 | 0.96 |
| 32 | 0.0025 | 0.96 |
| 33 | 0.0023 | 0.96 |
| 34 | 0.0023 | 0.96 |
| 35 | 0.0022 | 0.97 |
| 36 | 0.0019 | 0.97 |
| 37 | 0.0019 | 0.97 |
| 38 | 0.0019 | 0.97 |
| 39 | 0.0018 | 0.97 |
| 40 | 0.0018 | 0.98 |
| 41 | 0.0018 | 0.98 |
| 42 | 0.0017 | 0.98 |
| 43 | 0.0013 | 0.98 |
| 44 | 0.0013 | 0.98 |
| 45 | 0.0012 | 0.98 |
| 46 | 0.0010 | 0.98 |
| 47 | 0.0010 | 0.98 |
| 48 | 0.0010 | 0.99 |
| 49 | 0.00099 | 0.99 |
| 50 | 0.00095 | 0.99 |
| 51 | 0.00088 | 0.99 |
| 52 | 0.00087 | 0.99 |
| 53 | 0.00083 | 0.99 |
| 54 | 0.00075 | 0.99 |
| 55 | 0.00070 | 0.99 |
| 56 | 0.00067 | 0.99 |
| 57 | 0.00060 | 0.99 |
| 58 | 0.00052 | 0.99 |
| 59 | 0.00051 | 0.99 |
| 60 | 0.00047 | 0.99 |
| 61 | 0.00044 | 0.99 |
| 62 | 0.00044 | 1.00 |
| 63 | 0.00041 | 1.00 |
| 64 | 0.00041 | 1.00 |
| 65 | 0.00038 | 1.00 |
| 66 | 0.00036 | 1.00 |
| 67 | 0.00036 | 1.00 |
| 68 | 0.00033 | 1.00 |
| 69 | 0.00032 | 1.00 |
| 70 | 0.00027 | 1.00 |
| 71 | 0.00024 | 1.00 |
| 72 | 0.00023 | 1.00 |
| 73 | 0.00023 | 1.00 |
| 74 | 0.00020 | 1.00 |
| 75 | 0.00015 | 1.00 |
| 76 | 0.00015 | 1.00 |
| 77 | 0.00014 | 1.00 |
| 78 | 0.00013 | 1.00 |
| 79 | 0.00010 | 1.00 |
| 80 | 8.9 x 10^-05^ | 1.00 |
| 81 | 8.4 x 10^-05^ | 1.00 |
| 82 | 8.1 x 10^-05^ | 1.00 |
| 83 | 5.4 x 10^-05^ | 1.00 |
| 84 | 4.8 x 10^-05^ | 1.00 |
| 85 | 1.9 x 10^-05^ | 1.00 |
| 86 | 1.7 x 10^-05^ | 1.00 |
| 87 | 1.4 x 10^-05^ | 1.00 |
| 88 | 3.0 x 10^-05^ | 1.00 |
